# New Milk Protein-Derived Peptides with Potential Antimicrobial Activity: An Approach Based on Bioinformatic Studies

**DOI:** 10.3390/ijms150814531

**Published:** 2014-08-20

**Authors:** Bartłomiej Dziuba, Marta Dziuba

**Affiliations:** 1 University of Warmia and Mazury in Olsztyn, Chair of Industrial and Food Microbiology, Cieszynski Square 1, Olsztyn 10-957, Poland; 2 University of Warmia and Mazury in Olsztyn, Chair of Food Biochemistry, Cieszynski Square 1, Olsztyn 10-957, Poland; E-Mail: niklema@uwm.edu.pl

**Keywords:** milk proteins, *in silico* proteolysis, antimicrobial peptides, bioinformatics

## Abstract

New peptides with potential antimicrobial activity, encrypted in milk protein sequences, were searched for with the use of bioinformatic tools. The major milk proteins were hydrolyzed *in silico* by 28 enzymes. The obtained peptides were characterized by the following parameters: molecular weight, isoelectric point, composition and number of amino acid residues, net charge at pH 7.0, aliphatic index, instability index, Boman index, and GRAVY index, and compared with those calculated for known 416 antimicrobial peptides including 59 antimicrobial peptides (AMPs) from milk proteins listed in the BIOPEP database. A simple analysis of physico-chemical properties and the values of biological activity indicators were insufficient to select potentially antimicrobial peptides released *in silico* from milk proteins by proteolytic enzymes. The final selection was made based on the results of multidimensional statistical analysis such as support vector machines (SVM), random forest (RF), artificial neural networks (ANN) and discriminant analysis (DA) available in the Collection of Anti-Microbial Peptides (CAMP database). Eleven new peptides with potential antimicrobial activity were selected from all peptides released during *in silico* proteolysis of milk proteins.

## 1. Introduction

In evolutionary terms, milk is a model example of a molecular system containing substances with exceptional ability to prevent and inhibit microbial infections [1]. The role of molecules with preventive effects should be explored to promote the development of new antimicrobial treatments, new natural food preservatives or nutraceuticals [2]. The overall antimicrobial effectiveness of milk resulting from the synergistic activity of milk peptides and proteins other than immunoglobulins, such as lactoferrin, lactoperoxidase and lysozyme, is much higher than that of individual molecules [3]. Antimicrobial milk components may demonstrate antibiotic-like activity, and they could pose a natural alternative to antibiotics [4]. Moreover, the milk proteins’ sequences contain several motifs that can be released during enzymatic hydrolysis to increase antimicrobial potential of milk proteins. In the past 20 years, numerous proteins and peptides with antimicrobial properties have been isolated from various organisms and species, ranging from bacteria to humans, or released from food proteins by proteolysis [5,6,7,8]. Proteins with low molecular mass and peptides containing up to 80 amino acids, with highly varied structure, high specificity and activity, have been most thoroughly researched. Many of these peptides demonstrating potent antimicrobial activity are low-molecular mass peptides with up to 20 amino acid residues. Intensive works are being undertaken for isolating, purifying and describing peptides for commercial applications. More than 1000 linear peptides have been identified to date [9]. Their number continues to increase because peptides can be now isolated from sources other than living organisms, for instance from food proteins [10]. The new generation of native peptides is referred to collectively as antimicrobial peptides (AMPs).

Milk proteins are a natural source of bioactive peptides with diverse physiological and antimicrobial properties. Their activity is revealed after enzymatic proteolysis or after fermentation involving proteolytic microorganisms. Subject to proteolytic conditions, a diverse combination of bioactive peptides deriving from milk proteins can be obtained, but only few peptides have been identified and characterized for their antimicrobial activity [1,10,11,12,13,14,15,16]. Moreover, several milk protein-derived peptides demonstrate more than one type of activity, and they are referred to as multifunctional peptides [16,17].

A preliminary analysis of antimicrobial linear peptides of various origin and milk proteins revealed the presence of several motifs characterized by high structural similarity and, consequently, similar physicochemical properties and biological activity indicators. The above observation was used to formulate a research hypothesis that in addition to the identified fragments, milk protein sequences can contain several new motifs with antimicrobial activity.

The objective of this study was to search for new milk protein-derived peptides with antimicrobial potential applying computer simulated proteolysis of milk proteins [18] and prediction algorithms such as SVM (support vector machines), RF (random forest), ANN (artificial neural networks) and DA (discriminant analysis) available at the interface of the CAMP database [19].

## 2. Results and Discussion

### 2.1. Physico-Chemical Characteristic of AMPs

Comparison of theoretically calculated physicochemical properties and amino acid content of 416 antimicrobial peptides listed in BIOPEP database [20] with 59 antimicrobial peptides originating from milk proteins is summarized in Table 1 and Table 2. Physicochemical properties of individual AMPs from milk proteins are presented in Table S1. More than 80% of all peptides contain Lys, Gly and Leu amino acids. The Ile, Val, Ala, Arg, Ser, Phe, Asn, Thr, Gln and Pro are present in at least 50% of peptides, while Asp, Cys, Glu, His, Met, Trp and Tyr are their minor components. The content of amino acids in the sequences of the AMPs was calculated in reference to all of the examined peptides. The results indicate that certain amino acids are more common in the peptide sequences and can influence their biological activity. Amino acids such as Arg, His, Lys, Phe, Tyr, Trp, Leu, Pro could be predominant in the sequences of biologically active peptides, depending on their type of activity [21,22,23].

**Table 1:** Amino acid content of analyzed antimicrobial peptides.

Amino Acid	AMPs in BIOPEP				AMPs from Milk Proteins			
	Average Amino Acid Content (%)		Number of Peptides Containing Given Amino Acid	Min.-Max. Amino Acid Content (%)	Average Amino Acid Content (%)		Number of Peptides Containing Given Amino Acid	Min.-Max Amino Acid Content (%)
	a	b			a	b		
Ala	6.8	9.8	286	0–33.3	6.4	10.6	36	0–25.0
Arg	8.2	12.3	278	0–33.3	7.0	11.9	35	0–25.0
Asn	3.4	6.1	234	0–25.0	2.0	9.7	12	0–25.0
Asp	2.3	5.7	166	0–25.0	2.6	14.0	11	0–25.0
Cys	5.8	13.6	176	0–40.0	3.0	10.4	17	0–25.0
Gln	3.7	7.1	220	0–25.0	7.0	11.4	36	0–33.3
Glu	2.3	5.6	175	0–25.0	4.4	11.3	23	0–25.0
Gly	10.5	12.5	349	0–63.1	3.3	9.3	21	0–20.0
His	2.1	5.8	152	0–18.4	1.2	6.1	12	0–14.3
Ile	6.1	8.1	312	0–40.0	6.5	10.2	38	0–33.3
Leu	9.9	11.4	339	0–58.3	8.7	12.3	42	0–37.5
Lys	10.3	12.7	337	0–62.5	10.2	15.5	19	0–33.3
Met	1.0	3.7	113	0–7.4	1.0	3.6	16	0–7.1
Phe	4.0	6.2	258	0–37.5	2.6	7.3	21	0–16.7
Pro	5.0	9.7	215	0–53.2	8.0	11.8	40	0–28.6
Ser	5.1	7.5	282	0–23.1	4.0	9.1	26	0–21.4
Thr	3.4	6.3	226	0–33.3	5.3	10.8	29	0–33.3
Trp	2.1	5.0	178	0–38.5	3.2	8.0	24	0–16.7
Tyr	2.6	7.0	157	0–33.3	5.6	14.4	23	0–33.3
Val	5.9	8.2	300	0–37.3	8.0	12.5	38	0–37.5

a: For all antimicrobial peptides; and b: For peptides that contain given amino acid.

The highest amino acid content in all analyzed antimicrobial peptides was recorded for Lys, Gly, Arg and Leu, whereas the lowest content for Met, Trp, His, Asp, Glu and Tyr. However, evaluation of the amino acids content in the sequences where a given amino acid was found suggest that, in addition to the four predominant amino acids, Cys, Ala and Pro may also play a certain role. The majority of antimicrobial peptides are cationic amphiphatic peptides, containing one or no acidic residues and a high number of cationic Arg, Lys or His residues. Hydrophobic amino acids such as Trp, Val, Leu and Ileu account for 30%–50% of the peptide sequence and play a key role in secondary structure formation and interactions with bacterial membrane. The presence of cationic regions and the hydrophobic character of peptides influence the antibacterial activity [24,25]. Isracidin, the first antimicrobial cationic peptide derived from α_s1_-casein after chymosin cleavage, is a 23-mer peptide that contains 6 basic residues, including 5 in the *N*-terminal region, and 7 hydrophobic residues, with the net charge 2.2. Isracidin exhibits activity against a wide range of Gram-positive and Gram-negative bacteria [26]. The analysis of amino acid content of antimicrobial peptides revealed many fragments with the predominance of one or several amino acids [27,28].

In comparison with all AMPs listed in the BIOPEP database, amino acids such as Asn, Gly, Lys, Phe and Ser were less frequently encountered in AMPs from milk proteins. On average, the evaluated peptides had a higher content of Lys, Leu, Val and Pro. However, if the content of selected amino acids was evaluated only in sequences where they appeared, Asp, Glu, Tyr and Ala were encountered more frequently in several cases. The noted results are similar to those given by Wang and Wang [8] for AMPs in the Antimicrobial Peptide Database (APD database), but somewhat different from those reported by Hammami *et al.* [29]. The main difference relates to the presence of Ala residues in AMPs sequences in the Bactibase.

The specific properties of the peptide structure can be attributed to its amino acid composition. The minimum, mean and maximum values of selected physicochemical properties of the analyzed antimicrobial peptides are presented in Table 2.

**Table 2:** The physico-chemical characteristics of peptides collected in BIOPEP and peptides from milk proteins.

Index	AMPs Collected in BIOPEP			AMPs from Milk Proteins		
	Mean Value	Min.–Max. Value	Predominant Value (%)	Mean Value	Min.–Max. Value	Predominant Value (%)
Molecular mass (Da)	3242.9	393.5–14,350.8	2000–4000 (47)	1906.9	393.5–6707.4	393–1000 (39)
pI	9.3	3.4–13.3	9–10 (29)	8.1	3.4–12.0	10–11 (25)
Net charge	3.7	−7.0–20	0–5 (56)	2.2	−7.0–10.1	−2–0 (34)
Instability index	31.7	−50.4–166.2	0–60 (71)	41.5	−30.9–157.7	0–20 (24)
Aliphatic index	83.9	0–227.5	40–120 (65)	89.6	0.0–226.7	60–100 (49)
GRAVY	−0.2	−3.51–3.6	−1–0 (47)	−0.4	−2.5–2.2	−1–0 (49)
Boman Index (kcal/mol)	1.5	−2.6–6.8	1–2 (28)	1.3	−6.9–5.0	1–3 (61)

**Figure 1:**
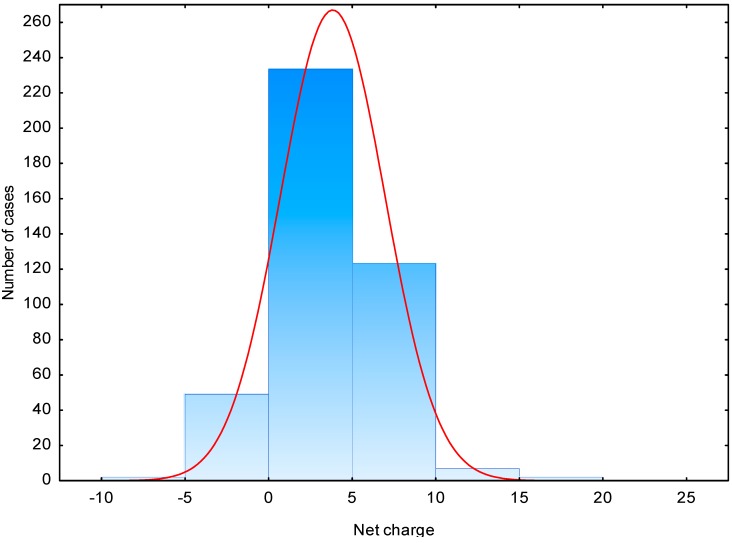
Net charge distribution for all antimicrobial peptides from BIOPEP database.

The molecular mass of the evaluated peptides ranged from 393 to 14,500 Da with an average of approximately 3200 Da. Molecules with a molecular mass in the 2000–4000 Da range were predominant in approximately 47% of all peptides. AMPs from milk proteins had lower molecular mass of 2000 Da on average, and they were characterized by a predominance of molecules with molecular mass under 1000 Da (approximately 39%). Out of 416 analyzed peptides, 57 had pI < pH 7 and 359 had pI > pH 7, whereas in the group of milk protein AMPs, 25 had pI < pH 7 and 34 had pI > pH 7. The above findings correlate with peptide net charges in neutral pH and the content of various amino acids discussed earlier. The net charge for all AMPs in BIOPEP ranged from −7.0 to 20.0. Thirty peptides were negatively charged, 344 had a positive charge, and the rest were neutral (Figure 1). The average net charge was 3.7, whereas the average net charge of peptides from the APD database was 4.56 [8]. In the analyzed group of milk protein AMPs, 12 were negatively charged, 35 had a positive charge and 12 were neutral. The average net charge was 2.2.

Many host-defense peptides that are produced naturally by the immune system have positively charged molecules, which can be attributed to the presence of basic amino acids in their sequence. The physical forces responsible for antibacterial activity are net positive charge, hydrophobicity and flexibility [22,30,31]. In addition to cationic AMPs, significant numbers of anionic peptides from eukaryotic organisms, including milk proteins, have been reported [1,27,32]. Anionic peptides are generally abundant in Asp and Glu, they have a net charge of −1 or −2, and similarly to cationic peptides, they can form amphiphilic structures, such as α-helix or β-sheet, that are crucial for their activity or exert various effects on sensitive microorganisms [1,25,33].

The value of the instability index calculated for 271 peptides (approximately 65%) and 30 milk protein peptides (51%) was below 40, and it was indicative of peptide stability [34]. The aliphatic index, which is positively correlated with thermostability, is defined as the relative volume of Ala, Val, Ile and Leu side chains. In eight analyzed fragments, the aliphatic index was zero, which implies an absence of the above amino acids in the analyzed peptides. The value of the aliphatic index was in the range of 40–120 for 65% of all peptides and 60–100 for 49% of milk protein AMPs. The value of the GRAVY index, a measure of peptide solubility, was negative for 232 (56%) AMPs in the BIOPEP database and 30 milk protein AMPs (51%), and it was indicative of their hydrophilic nature [35]. The value of the Boman index, which is a measure of peptide affinity to proteins and its ability to establish biological interactions, ranged from −2.6 to 6.8 for all analyzed peptides and from −6.9 to 5.0 for milk protein AMPs. The predominant value of the Boman index was 1–2 for all AMPs (28%) and 1–3 for milk protein AMPs (61%).

The physicochemical properties of peptides and their biological activity indicators were used to select new potentially antimicrobial peptides released *in silico* from milk proteins. However, the main criterion of performed prediction was the analysis made with statistical tools available in CAMP database [36].

### 2.2. In Silico Proteolysis of Milk Proteins

**Figure 2:**
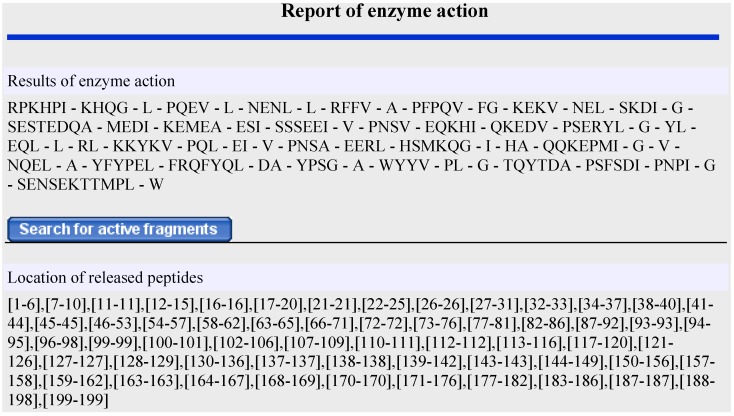
Results window of peptides released from bovine α_s1_-casein var. gen. B by pancreatic elastase (EC 3.4.21.36) generated by BIOEP database.

Several strategies are applied to identify and produce biologically active peptides from milk proteins [37]. At first, peptides present in the protein sequence have to be released by enzymatic hydrolysis with digestive enzymes [27,38,39,40], by fermenting milk with proteolytic starter cultures [41,42,43] or by the action of enzymes derived from microorganisms [12,44]. Proteolytic enzymes, such as pepsin, trypsin, alcalase, chymotrypsin, papain and pancreatic elastase, are most commonly applied to obtain bioactive peptides [24,45]. In the next stage, peptide fractions are separated and purified, their antimicrobial properties are determined, and they are identified usually by various MS techniques. Due to the fact that sequences of milk proteins and the cleavage sites of enzymes are known as well as the properties of antimicrobial peptides are well defined it is possible to apply bioinformatic tools in the search for new AMPs. This strategy is based on computer simulation of proteolysis and application of multivariate statistical methods to determine potentially antimicrobial motifs released from the analyzed proteins. *In silico* proteolysis can be performed with the use of several programs, such as PMAP (www.proteolysis.org/proteases) [46] and PeptideCutter (www.expasy.org/tools/peptidecutter/) [47], or a dedicated tool in the BIOPEP database. Detailed description of BIOPEP database has been presented in our earlier work [18]. Briefly, the “Record Operation” menu in BIOPEP contains the “Enzyme Action” option, which can be used to design the proteolytic process. In this study, 28 enzymes from the BIOPEP database were used to simulate the proteolysis of major milk proteins. The simulation produced thousands of milk protein fragments. Antimicrobial activity predictions were based solely on peptide chains containing 5 to 30 amino acids. The results example of *in silico* proteolysis of α_s1_-casein var. gen. B by pancreatic elastase is presented in Figure 2. Only 15 out of 58 fragments were further evaluated.

Additionally, the “search for active fragments” command was used to determine released fragments whose structure corresponded to that of peptides with known antimicrobial activity. Those fragments were not further examined.

### 2.3. Prediction of Antimicrobial Activity of Peptides Released During in Silico Proteolysis of Milk Proteins

The antimicrobial activity of peptides can be analyzed with the use of dedicated tools in several databases. Various search algorithms are available in APD [8,48], Bactibase [29], PhytAMP [49], AntiBP [50], AMPA [51] and CAMP [19,52]. APD relies on the physicochemical properties of peptides, and if those parameters are within the APD-defined space for natural AMPs, the program will align their sequences with those present in the database. PhytAMP and Bactibase use the Hidden Markov model for the sequence alignment. Above two databases are mainly dedicated to plant AMPs and bacteriocins, respectively. AntiBP analyses introduced sequences using three statistical methods: Artificial Neural Networks, Support Vector Machines and Quantitive Matrices. Prediction models are dedicated to peptides longer than 15 amino acids. The prediction tool available in AMPA is a highly interesting option. The AMPA algorithm uses the propensity scale to generate an antimicrobial profile. The calculation is based on antimicrobial indices defined for individual residues. The AMPA algorithm is useful in analyses of whole proteins rather than their fragments. The prediction tools available in CAMP, based on machine learning algorithms such as SVM, RF, ANN and DA, were most suitable for our research needs. According to Thomas *et al.* [19], the results accuracy for different statistical models ranges from 87% to 93%. The discussed tools can be used to predict the antimicrobial activity of peptides regardless of their length.

In the first stage, the antimicrobial potential of milk protein fragments obtained by proteolysis was evaluated with the use of four statistical models available in the CAMP database. The results were used to manually select peptides where a positive result was reported in at least two algorithms. Overlapping peptides were excluded based on sequences of known AMPs from milk proteins. The above procedure supported the identification of 60 fragments whose sequences and properties are presented in Table S2. In the following stage, peptides whose sequences partially overlapped the sequences of the identified AMPs as well as peptides with low water solubility, as predicted by the peptide property calculator [53] (http://www.innovagen.com), were eliminated. A total of 11 potentially antimicrobial peptides with the prediction scores higher than 0.45 for at least three statistical models (Table 3) were thus selected. The sequences of potentially antimicrobial AMPs were not located nearby the sequences of the known AMPs. The peptides identified in CAMP were additionally analyzed with the use of the tools available in the APD database.

**Table 3:** The characteristics of potential AMPs from milk proteins released during *in silico* proteolysis and predicted by statistical models available in CAMP database (SVM, RF, ANN and DA).

Sequence	AMP Origin/Position	Net Charge	Isoelectric Point pH	Molecular Mass (Da)	Boman Index	Instability Index	Aliphatic Index	GRAVY	SVM ^a^	RFC ^b^	ANN ^c^	DAC ^d^
DDKHYQKA (Pancreatic elastase EC 3.4.21.36, Leukocyte elastase EC 3.4.21.37)	α_s2_-casein, gen. var. A-11P f(74–81)	0.1	7.72	1004.07	4.63	53.06	12.50	−2.625	1.000	0.622	NAMP	0.507
GQRDLLFKDSALGFLRIP (Prolyl oligopeptidase EC 3.4.21.26)	Lactoferrin f(294–311)	1.0	10.08	2046.41	1.52	20.89	113.89	0.028	0.659	0.4835	AMP	0.834
ADALNLDGGYIYTAGKCGLVPVLAE (V-8 protease EC 3.4.21.19)	Lactoferrin f(389–413)	−2.0	3.7	2523.89	−0.22	16.36	117.20	0.536	0.568	0.5215	NAMP	0.816
QEQNQEQP (Prolyl oligopeptidase EC 3.4.21.26), Thermolysin EC 3.4.24.27)	κ-casein, gen. var. A f(1–8)	−2.0	3.79	999.9	5.3	119.70	0.00	−3.263	0.976	0.5845	AMP	0.000
KKYKVPQL (Pepsin 1.3 EC 3.4.23.1, Pancreatic elastase EC 3.4.21.71)	α_s1_-casein, gen. var. B-8P f(102–109)	3.0	10.45	1003.25	1.67	46.29	85.00	−1.262	0.952	0.509	NAMP	0.916
AVAVVKKGSNF (Chymase EC 3.4.212.39, Metridin EC 3.4.21.3)	Lactoferrin f(94–104)	2.0	10.6	1119.33	0.13	−14.91	97.27	0.591	0.943	0.483	AMP	0.912
EMPFPK(Ficain EC 3.4.22.3, Bromelain EC 3.4.22.4)	β-casein, gen. var. A^2^-5P f(108–113)	0.0	6.94	747.91	1.17	145.77	0.00	−0.983	1.000	0.571	AMP	0.190
EPEQSL (Ficain EC 3.4.22.3)	β-lactoglobulin gen. var. B f(112–117)	−2.0	3.13	701.73	2.94	174.73	65.00	−1.517	1.000	0.4695	NAMP	0.569
ITRINKKIEKFQS (Leukocyte elastase EC 3.4.21.37)	β-casein, gen. var. A^2^-5P f(23–35)	3.0	10.83	1604.92	2.98	88.52	90.00	−0.915	0.492	0.45	AMP	0.739
ITRINKKIEKF (Proteinase P1 (lactocepin) EC 3.4.21.96)	β-casein, gen. var. A^2^-5P f(23–33)	3.0	10.83	1389.71	2.71	62.85	106.36	−0.691	0.694	0.46	AMP	0.879
ALFGKNGKNCPDKFCLFK (Proteinase P1 (lactocepin) EC 3.4.21.96)	Lactoferrin f(616–633)	2.9	9.71	2030.45	1.06	−5.79	48.89	−0.317	0.706	0.7655	AMP	0.987

^a^, SVM: support vector machines; ^b^, RF: random forest; ^c^, ANN: artificial neural networks; and ^d^, DA: discriminant analysis.

In the group of 28 enzymes applied in *in silico* proteolysis, 11 of them released fragments whose antimicrobial potential was predicted in the described procedure. Four potentially antimicrobial AMPs were released from lactoferrin by chymase, metridin, prolyl oligopeptidase, proteinase P1 and V-8 protease, three from β-casein by bromelain, ficain, leukocyte elastase, proteinase P1, and one each from the remaining casein fractions by leukocyte elastase, pancreatic elastase, pepsin, prolyl oligopeptidase and thermolysin, and one from β-lactoglobulin by ficain. Low-molecular mass peptides in the range of 390–2500 Da were the predominant group of milk protein-derived peptides with antimicrobial properties (73%). The above observations were confirmed by the results of extensive research into milk proteins, in particular with regard to isracidin (α_s1_-casein f(7–42)) [26]. Four smaller fragments with antimicrobial activity were derived from isracidin: caseicin A and B [12], fragment 1–7 and fragment 10–14 [43]. The selected in our work, potentially antimicrobial AMPs contained 5 to 25 amino acids, which corresponds to molecular mass of 701 to 2520 Da.

The general classification of AMPs include cationic peptides which can be divided in three subclasses: linear peptides forming helical structures, cysteine-rich open-ended peptides containing single or several disulfide bridges and molecules rich in specific amino acids, such as proline, glycine, histidine and tryptophan [54]. Peptides with a positive charge constitute the majority of antimicrobial peptides known as cationic antimicrobial peptides (CAPs). In the group of known AMPs derived from milk proteins (Table S1), 35 have a positive charge ranging from 0.1 to 10.1. The second group contains anionic peptides that are generally rich in glutamic and aspartic acid [54]. Those peptides account for approximately 30% of all known milk protein AMPs. Another important feature characterizing AMPs is their ability to form amphipathic structures [24]. This process involves hydrophobic amino acids such as tryptophan and valine, which account for 30%–50% of the peptide sequence [22]. An analysis of selected peptides with the use of APD tools revealed that five fragments can form amphipathic helices. Fragment GQRDLLFKDSALGFLRIP (Table 3) contains three cationic and two anionic residues, two arginines, one proline, and it may have five residues on the same hydrophobic surface. Peptides ITRINKKIEKFQS and ITRINKKIEKF have four positively charged and one negatively charged residue, and they may have three residues on the same hydrophobic surface. Peptide ALFGKNGKNCPDKFCLFK has four positively charged and one negatively charged residue and, additionally, two cysteine residues that could form a disulfide bond stabilizing the α helix or the beta structure. The negatively charged ADALNLDGGYIYTAGKCGLVPVLAE peptide has three negatively charged residues (two aspartic acids and one glutamic acid), one positively charged residue, one proline and, possibly, five residues on the same hydrophobic surface. The AVAVVKKGSNF fragment is cationic due to the presence of two lysines with a high hydrophobic ratio of 54%, but only two residues have been predicted on the same hydrophobic surface. The remaining five peptides were very short, ranging from six to eight residues. According to Laverty *et al.* [23] and Strøm *et al.* [55], ultra short antimicrobial peptides consist of approximately four or five amino acid residues with a selection of amino acids that fulfill minimum functionalities required for effective antimicrobial activity. Those functionalities include charged moieties, such as arginine and the lipophilic tryptophan, which form an antimicrobial pharmacophore with the correct balance between charge and lipophilicity. In view of the above, the prediction scores for the five shortest peptides—DDKHYQKA, QNQNQEP, KKYKVPQL, EMPFPK and EPEQSL—are difficult to explain. According to the APD database, those peptides are too short to form a helix, and they do not have residues on the same hydrophobic surface. Two of them are neutral, two are anionic and one is cationic. Fragment DDKHYQKA contains three positively charged and two negatively charged residues, but due to the presence of glutamine, the overall charge is 0.1. Fragment QEQNQEQP contains proline and two glutamic acids residues typical for anionic peptides. The hydrophobic ratio for both fragments is low at 12% and 0%, respectively. Fragment EMPFPK is a neutral peptide with one positively charged and one negatively charged residue, but it also contains two prolines, and its hydrophobic ratio is 33%. Peptide EPEQSL has two negatively charged residues, it contains one proline and has a hydrophobic ratio of 16%. Fragment KKYKVPQL contains three positively charged lysines, a proline residue, and its hydrophobic ratio is 25%. However, it is worth noting that some small peptides derived from milk proteins, such as GLPQE, EQLTK, STVATL and YVL, which also do not form amphipathic structures, exhibit antimicrobial activity. Several peptides are known to impact more than one physiological function. Small AMPs derived from bovine κ-casein YLV f(30–32), IQD f(28–30) and ovine α_S2_-casein LKKISQ f(165–170), PYVRYL f(203–208) also demonstrate antioxidative and ACE-inhibitory activity, respectively [56]. The mechanism of action of short peptides has not been fully explained and may be linked to a different mode of action [57].

Stability is yet another important feature of AMPs. Molecules with a stability index higher than 40 are regarded as unstable, and therefore they are characterized by lower bioavailability and shorter half-life. In the group of selected peptides (Table 3), six were predicted to be stable. Some processing techniques, such as chemical modification or incorporation of synthetic amino acids, can be applied to increase peptide stability and, consequently, lower susceptibility to hydrolysis by proteases. In the peptide property calculator application [53] (http://www.innovagen.com), many peptides that had been predicted to be antimicrobial were determined as non-soluble due to their high hydrophobicity. Selected examples, characterized by high prediction scores in the applied statistical models, include LF f(149–175) AVAKFFSASCVPCIDRQAYPNLCQLCKG, LF f(147–153) GAVAKFF, LF f(60–65) DGGMVF, LF f(124–137) GWIIPMGILRPYLS, LF f(432–443) GYLAVAVVKKAN, LF f(666–686) VTAIANLKKCSTSPLLEACAF, LF f(126–135) IIPMGILRPY, LF f(120–132) GRSAGWIIPMGIL, LF f(517–525) LQGAVAKFFSASCVP, κ-casein, gen. var. A f(57–63) PYPYYAK. One of the drawbacks associated with the application of such AMPs is low solubility, which inhibits peptide delivery to target cells and tissues and peptide transport across membranes. The solubility problem may be addressed by gentle heating with sonification or through the application of DMSO (dimethyl sulfoxide), which is often used in pharmacology [58]. Delivery issues may be resolved through encapsulation in coordination polymers or particles and the use of micro- or nano-sized liquid marbles. Possible solutions to inhibited peptide transport across membranes include the development of soluble pro-drugs, the use of liquid dispersions and encapsulation in liposomes [44,45].

## 3. Experimental Section

### 3.1. Materials

Milk proteins as potential precursors of new antimicrobial peptides (AMPs) and their physicochemical properties were evaluated based on major milk proteins and AMPs listed in the BIOPEP database. The amino acid sequences of reference proteins for α_s1_-, α_s2_-, β-, κ-casein, β-lactoglobulin, α-lactalbumin, lactoferrin and 416 AMPs were analyzed.

### 3.2. Physicochemical Properties of AMPs

The physicochemical properties of antimicrobial peptides listed in the BIOPEP database were calculated with the use of software and algorithms freely available on the internet. Molecular mass, isoelectric point, the number and composition of amino acid residues, values of the instability index, aliphatic index and GRAVY index (grand average of hydropathicity) of antimicrobial peptides were computed in the ProtParam application [59]. The Boman index was calculated with APD2 algorithm: Antimicrobial Peptide Calculator and Predictor [60], and net charge was determined using Innovagen’s Peptide Property Calculator [53].

### 3.3. In Silico Proteolysis of Milk Proteins

The amino acid sequences of reference milk proteins were subjected to *in silico* proteolysis in the BIOPEP database [20]. The “Enzyme(s) action” application was used to determine the release of peptides from precursor proteins. The above supported the hydrolysis of proteins with up to three randomly-selected proteolytic enzymes out of the 28 available in the database. The option involving a single enzyme was applied for protein hydrolysis *in silico* in this experiment. Released peptides composed of 5 to 30 amino acid residues were submitted to statistical prediction of antimicrobial activity.

### 3.4. Prediction of Antimicrobial Activity of Peptides Released during in Silico Proteolysis of Milk Proteins

The antimicrobial activity of the released peptides was determined with the use of the Prediction Antimicrobial Peptides tool in the CAMP database [19,36]. Four multivariate statistical methods were used for prediction: Random Forest (RF), Support Vector Machines (SVM), Artificial Neural Network (ANN) and Discriminant Analysis (DA). The process of model development and evaluation has been described in detail by Waghu *et al.* [52]. Prediction results are presented with the relevant scores, excluding ANN, and peptides are classified as AMPs or non-AMPs. In this study, peptides were classified as AMPs if the resulting score was higher than 0.45 and if a positive recognition was obtained for at least three statistical methods. The selected peptides were processed in the APD database to describe their amino acid content and structure and to determine the presence of residues on the same hydrophobic surface of the molecules [48].

## 4. Conclusions

Research into bioactive peptides expands our knowledge of correlations between heath and diet. Increasing attention has been focused on AMPs due to their potential as a novel therapeutics. The results of the study indicate that milk proteins can be a source of new, potentially antimicrobial peptides. The bioinformatics applications that proved to be most useful for the needs of our research were the AMP prediction tools in the CAMP database that rely on machine learning algorithms such as SVM, RF, DA and ANN. Our results suggest that major milk proteins contain 60 potential AMPs that can be released by proteolytic enzymes. The highest prediction scores were reported for 11 potential AMPs in at least three statistical models. Those peptides that can be released by bromelain, chymase, ficain, leukocyte elastase, metridin, pancreatic elastase, pepsin, prolyl oligopeptidase, proteinase P1 thermolysin, V-8 protease were selected for chemical synthesis and further *in vitro* bioactivity analyses, which would verify the validity of the bioinformatics approach proposed in this study.

## Supplementary Files

Supplementary File 1
