# Supplementary material for: New Milk Protein-Derived Peptides with Potential Antimicrobial Activity: An Approach Based on Bioinformatic Studies
Source: Int J Mol Sci. 2014 Aug 20;15(8):14531–45. doi: 10.3390/ijms150814531 (PMC4159866; doi:10.3390/ijms150814531)
Supplement: Supplementary File 1 [file ijms-15-14531-s001.pdf]

Supplementary Information

Table S1. The characteristics of known AMPs derived from milk proteins.

Sequence	AMP Origin/Position	Net Charge	Isoelectric Point (pH)	Molecular Mass (Da)	GRAVY	Instability Index	Aliphatic Index	Half Life	Boman Index (kcal/mol)
RPKHPIK	f(1–7) of α_{S1}-casein	3.1	11.17	875.0	−2.029	19.84	55.71	Mamalian 1 h<br>Yeast 2 min<br>E.coli 2 min	3.68
RPKHPIKHQGLPQEVL NENLLRF	Isracidin f(1–23) of α_{S1}-casein	2.2	9.99	2764.2	−0.987	46.49	97.39	Mamalian 1 h<br>Yeast 2 min<br>E.coli 2 min	2.42
GLPQE	f(10–14) of α_{S1}-casein	−1.0	4.00	542.5	−1.040	123.56	78.00	Mamalian 10 h<br>Yeast >20 h<br>E.coli >20 h	1.29
IKHQGLPQE	f(6–14) of α_{S1}-casein	0.1	6.75	1049.1	−1.256	63.66	86.67	Mamalian 20 h<br>Yeast 30 min<br>E.coli >10 h	1.92
VLNENLLR	f(15–22) of α_{S1}-casein	0.0	5.97	970.1	0.075	32.83	182.5	Mamalian 100 h<br>Yeast >20 h<br>E.coli >10 h	2.02
LRLKKYKVPQL	α_{S1}-casein, gen. var. B-8P f(99–109)	4	10.46	1385.7	−0.636	46.18	132.73	Mamalian 5.5 h<br>Yeast 3 min<br>E.coli 2 min	1.67
SDIPNPIGSENSEK	f(180–193) of α_{S1}-casein	−2	4.14	1486.5	−1.314	32.69	55.71	Mamalian 1.9 h<br>Yeast >20 h<br>E.coli >10 h	2.89
KTKLTEEEKNRLNFLK KISQRYQKFALPQYLK TVYQHQK	casocidin-I f(150–188) of α_{S2}-casein gen. var. A	7.1	10.08	4869.6	−1.323	27.85	70.00	Mamalian 1.3 h<br>Yeast 3 min<br>E.coli 2 min	2.83
VYQHQKAMKPWIQPK TKVIPYVRY	f(183–206) α_{S2}-casein gen. var. A	5.1	10.12	3002.6	−0.783	2.25	72.92	Mamalian 100 h<br>Yeast >20 h<br>E.coli >10 h	1.37
LKKISQ	f(165–170) of ovine α_{S2}-casein	2.0	10.00	715.8	−0.633	12.13	130.00	Mamalian 5.5 h<br>Yeast 3 min<br>E.coli 2 min	1.7
VYQHQKAMKPWIQPK TKVIPYVRYL	f(183–207) of bovine α_{S2}-casein	5.1	10.12	3115.7	−0.600	2.56	85.60	Mamalian 100 h<br>Yeast >20 h<br>E.coli >10 h	1.12

Table S1. *Cont.*

Sequence	AMP Origin/Position	Net Charge	Isoelectric Point (pH)	Molecular Mass (Da)	GRAVY	Instability Index	Aliphatic Index	Half Life		Boman Index (kcal/mol)
LKKISQRYQKFALPQY	f(164–179) of bovine α_{S2}-casein	4.0	10.17	2011.4	−0.938	25.45	79.38	Mamalian<br>Yeast<br>E.coli	5.5 h<br>3 min<br>2 min	2.02
PYVRYL	f(203–208) of ovine α_{S2}-casein	1.0	9.00	809.9	−0.117	−4.23	113.33	Mamalian<br>Yeast<br>E.coli	>20 h<br>>20 h<br>?	1.04
KTVYQHQKAMKPWIQ PKTKVIPYVRYL	f(181–207) of ovine α_{S2}-casein	6.1	10.22	3345.0	−0.726	3.11	79.26	Mamalian<br>Yeast<br>E.coli	1.3 h<br>3 min<br>3 min	1.33
LKTVYQHQKAMKPWI QPKTKVIPYVRYL	f(180–207) of ovine α_{S2}-casein	6.1	10.22	3458.22	−0.564	0.32	90.36	Mamalian<br>Yeast<br>E.coli	5.5<br>3min<br>3min	1.11
ALPQYLKTVYQHQKA MKPWIQPKTKVIPYVR YL	f(175–207) of ovine α_{S2}-casein	6.1	10.1	4030.8	−0.503	11.18	91.52	Mamalian<br>Yeast<br>E.coli	4.4 h<br>>20 min<br>>10 min	0.91
QKFALPQYLKTVYQHQ KAMKPWIQPKTKVIPY VRYL	f(172–207) of ovine α_{S2}-casein	7.1	10.17	4434.3	−0.589	11.08	83.89	Mamalian<br>Yeast<br>E.coli	0.8 h<br>10 min<br>10 h	1.06
LKKISQRYQKFALPQY LKTVYQHQKAMKPWI QPKTKVIPYVRYL	f(164–207) of ovine α_{S2}-casein	10.1	10.35	5451.6	−0.700	9.69	86.36	Mamalian<br>Yeast<br>E.coli	5.5 h<br>2 min<br>2 min	1.44
LKKISQRYQKFALPQ YL	f(164–180) of ovine α_{S2}-casein	4.0	10.17	2124.5	−0.659	24.54	97.65	Mamalian<br>Yeast<br>E.coli	5.5 h<br>2 min<br>2 min	1.61
YQEPVLGPVRGPFPI	β-casein, gen. var. A^2-5P f(193–207)	0	6.00	1668.9	−0.033	80.71	90.67	Mamalian<br>Yeast<br>E.coli	2.8 h<br>10 min<br>2 min	0.3
YQEPVLGPVRGPFPIIV	β-casein, genetic variant A^2-5P f(193–209)	0	6.00	1881.2	0.482	67.4	120.00	Mamalian<br>Yeast<br>E.coli	2.8 h<br>10 min<br>2 min	−0.25
MAIPPKKNQDKTEIPTI NTIASGEPTSTPTTEAV ESTVATLEDSPEVIESPP EINTVQVTSTAV	Kappacin f(107–169) of κ-casein gen. var. A	−7.0	4.04	6707.4	−0.370	64.17	77.66	Mamalian<br>Yeast<br>E.coli	30 h<br>20 h<br>10 h	1.55

Table S1. *Cont.*

Sequence	AMP Origin/Position	Net Charge	Isoelectric Point (pH)	Molecular Mass (Da)	GRAVY	Instability Index	Aliphatic Index	Half Life		Boman Index (kcal/mol)
YYQQKPVA	f(42–49) of bovine κ-casein	1.0	8.50	996.1	−1.137	62.9	48.75	Mamalian<br>Yeast<br>E.coli	2.8 h<br>10 min<br>2 min	1.38
VQVTSTAV	f(162–169) of bovine κ-casein	0.0	5.49	803.9	1.087	−11.29	121.25	Mamalian<br>Yeast<br>E.coli	100 h<br>>20 h<br>>10 h	0.01
STVATL	f(141–146) of bovine κ-casein	0.0	5.24	590.6	1.267	8.33	130.00	Mamalian<br>Yeast<br>E.coli	1.9 h<br>>20 h<br>>10 h	−0.37
FSDKIAK	f(18–24) of bovine κ-casein	1.0	8.59	807.9	−0.429	−15.69	70.00	Mamalian<br>Yeast<br>E.coli	1.1 h<br>3 min<br>2 min	1.93
IQY	f(28–30) of bovine κ-casein	0.0	6.09	422.79	−0.100	−18.47	130.00	Mamalian<br>Yeast<br>E.coli	20 h<br>>30 min<br>>10 h	0.25
YVL	f(30–32) of bovine κ-casein	0.0	6.09	393.51	2.233	6.67	226.67	Mamalian<br>Yeast<br>E.coli	20 h<br>30 min<br>>10 h	−2.59
EIPT	f(118–121) of bovine κ-casein	−1.0	3.85	458.54	−0.325	3.85	97.50	Mamalian<br>Yeast<br>E.coli	1 h<br>30 min<br>>10 h	1.11
VESTVATL	f(139–146) of bovine κ-casein	−1.0	4.00	818.9	1.038	32.83	133.75	Mamalian<br>Yeast<br>E.coli	100 h<br>>20 h<br>>10 h	0.08
PAAVRSPAQILQ	f(64–75) of bovine κ-casein	1.0	10.18	1250.4	0.200	157.72	114.17	Mamalian<br>Yeast<br>E.coli	>20 h<br>>20 h<br>?	0.84
AVESTVATLEDSPEVIE SPPE	f(136–156) of bovine κ-casein	−6.0	3.40	2199.3	−0.243	134.70	88.10	Mamalian<br>Yeast<br>E.coli	4.4 h<br>>20 h<br>>10 h	1.54
EQLTK	LTD1-f(1–5) of α-laktoalbumin, gen. var. B	0.0	6.10	617.7	−1.560	46.52	78.00	Mamalian<br>Yeast<br>E.coli	1h<br>30 min<br>>10 h	3.11

Table S1. *Cont.*

Sequence	AMP Origin/Position	Net Charge	Isoelectric Point (pH)	Molecular Mass (Da)	GRAVY	Instability Index	Aliphatic Index	Half Life		Boman Index (kcal/mol)
GYGGVSLPEWVCTTF	LDT 2, α-chain f(17–31) of α- laktoalbumin, gen. var. B	−1.0	4.00	1615.8	0.453	44.95	64.67	Mamalian<br>Yeast<br>E.coli	>30 h<br>>20 h<br>>10 h	−0.46
ALCSEK	LDT 2, β-chain f(109–114) of α-laktoalbumin, . gen. varB	0.0	6.04	649.7	−0.017	40.43	81.67	Mamalian<br>Yeast<br>E.coli	4.4 h<br>>20 h<br>>10 h	1.29
CKDDQNPH	LDC, α-chain f(61–68) of α-laktoalbumin, gen. var. B	−1.0	5.21	956.0	−2.525	5.15	0.00	Mamalian<br>Yeast<br>E.coli	1.2 h<br>>20 h<br>>10 h	4.81
ISCDKF	LDC, β-chain chain f(75–80) of α-laktoalbumin, gen. var. B	0.0	5.83	711.8	0.267	80.62	65.00	Mamalian<br>Yeast<br>E.coli	20 h<br>30 min<br>>10 h	1.41
VAGTWY	f(15–20) β-laktoglobulin, gen. var. A	0.0	5.49	695.7	0.450	−30.87	65.00	Mamalian<br>Yeast<br>E.coli	100 h<br>>20 h<br>>10 h	−1.06
IPAVFK	f(78–83) β-laktoglobulin, gen. var. A	1.0	8.75	673.8	1.300	10.58	130.00	Mamalian<br>Yeast<br>E.coli	20 h<br>30 min<br>>10 h	−1.36
AASDISLLDAQSAPLR	f(25–40) β-laktoglobulin, gen. var. A	−1.0	4.21	1627.8	0.256	60.91	122.50	Mamalian<br>Yeast<br>E.coli	4.4 h<br>>20 h<br>>10 h	1.32
VLVLDTDYK	f(92–100) β-laktoglobulin, gen.var. A	−1.0	4.21	1065.2	0.344	−7.81	151.11	Mamalian<br>Yeast<br>E.coli	100 h<br>>20 h<br>>10 h	0.86
FKCRRWQWRMKKLG APSITCVRRAF	bovine lactoferricin B f(17–41) of lactoferrin	7.9	11.84	3125.8	−0.576	77.92	50.80	Mamalian<br>Yeast<br>E.coli	1.1 h<br>3 min<br>2 min	2.75
APRKNVRW	f(1–8) of lactoferrin	3.0	12.01	1026.2	−1.613	95.00	48.75	Mamalian<br>Yeast<br>E.coli	4.4 h<br>>20 h<br>>10 h	4.23
FKCRRWQWRMKKLG APSITCVRRAFA	f(17–42) of lactoferrin	7.9	11.84	3196.9	−0.485	75.31	52.69	Mamalian<br>Yeast<br>E.coli	1.1 h<br>3 min<br>2 min	2.57

Table S1. *Cont.*

Sequence	AMP Origin/Position	Net Charge	Isoelectric Point (pH)	Molecular Mass (Da)	GRAVY	Instability Index	Aliphatic Index	Half Life		Boman Index (kcal/mol)
FKCRRWQWRMKKLGAPSITCVR RAFAL	f(17–43) of lactoferrin	7.9	11.84	3310.0	−0.326	72.89	65.19	Mamalian<br>Yeast<br>E.coli	1.1 h<br>3 min<br>2 min	2.29
APRKNVRWCTISQPEW	f(1–16) of lactoferrin	2.0	9.51	1971.2	−1.056	98.42	48.75	Mamalian<br>Yeast<br>E.coli	4.4 h<br>>20 h<br>>10 h	2.72
CIRA	f(45–48) of lactoferrin	1.0	8.55	461.61	1.075	7.50	122.5	Mamalian<br>Yeast<br>E.coli	1.2 h<br>>20 h<br>>10 h	3.69
FKCRRWQWRMKKLGAPSITCVR RAFALECIR	f(17–47) of lactoferrin	7.9	11.37	3811.6	−0.316	78.95	69.35	Mamalian<br>Yeast<br>E.coli	1.1 h<br>3 min<br>2 min	2.5
APRKNVRWCTI	f(1–11) of lactoferrin	3.0	10.86	1343.6	−0.600	101.45	70.91	Mamalian<br>Yeast<br>E.coli	4.4 h<br>>20 h<br>>10 h	2.74
CRRWQWRMKKLGAPSITCV	f(19–37) of lactofferin	4.9	10.93	2319.8	−0.468	77.14	61.58	Mamalian<br>Yeast<br>E.coli	1.2 h<br>>20 h<br>>10 h	2.16
FKCRRWQWRMKKLG	f(17–30) of lactofferin	6.0	11.74	1923.3	−1.421	74.31	27.86	Mamalian<br>Yeast<br>E.coli	1.1 h<br>3 min<br>2 min	3.55
LECIRA	f(43–48) of lactofferin	0.0	5.99	703.8	0.767	81.57	146.67	Mamalian<br>Yeast<br>E.coli	5.5 h<br>3 min<br>2 min	1.46
APRKNVRWCTISQPEWFKCRRW QWRMKKLGAPSITCVRRAFA	f(1–42) of lactofferin	9.9	11.63	5150.1	−0.702	84.35	51.19	Mamalian<br>Yeast<br>E.coli	4.4 h<br>>20 h<br>>10 h	2.63
FKCRRWQWRMKKLGAPSITCVR RAFALECIRA	f(17–48) of lactofferin	7.9	11.37	3883.7	−0.250	76.80	70.31	Mamalian<br>Yeast<br>E.coli	1.1 h<br>3 min<br>2 min	2.36
WKLLSKAQEKFGKNKSR	Lactoferrampin f(268–284) of lactofferin	5.0	10.58	2048.4	−1.482	36.01	51.73	Mamalian<br>Yeast<br>E.coli	2.8 h<br>3 min<br>2 min	2.97

Table S1. *Cont.*

Sequence	AMP Origin/Position	Net Charge	Isoelectric Point (pH)	Molecular Mass (Da)	GRAVY	Instability Index	Aliphatic Index	Half Life		Boman Index (kcal/mol)
ADRDQYELL	Antiviral f(241–249) of lactofferin	−2.0	4.03	1122.2	−1.156	−25.68	97.78	Mamalian<br>Yeast<br>E.coli	4.4 h<br>>20 h<br>>10 h	3.68
TRVVWCAVG	Lactoferrin (fr. 343–351)	1	9.01	990.1	1.156	−0.54	107.78	Mamalian	7.2 h	−0.11
GRDPYKLRPV	Lactoferrin (fr. 68–77)	2	9.99	1200.4	−1.33	58.29	68.00	Yeast	>20 h	3.43
EDLIWK	Antiviral f(263–268) of lactofferin	−1.0	4.37	802.9	−0.583	40.43	130.00	Mamalian<br>Yeast<br>E.coli	1 h<br>30 min<br>>10 h	1.48

Table S2. The characteristics of potential AMPs from milk proteins released during in silico proteolysis according to positive prediction scores of two or more statistical models available in CAMP database (SVM, RF, ANN and DA).

Sequence (Enzyme)	AMP Origin/Position	Net Charge	Isoelectric Point pH	Molecular Mass Da	Boman Index kcal/mol	Instability Index	Aliphatic Index	GRAVY	SVM	RFC	ANN	DAC
DDKHYQKA (Pancreatic elastase EC 3.4.21.36, Leukocyte elastase EC 3.4.21.37)	α_{s2}-casein, gen. var. A-11P f(74–81)	0.1	7.72	1004.07	4.63	53.06	12.50	−2.625	1.000	0.622	NAMP	0.507
FTKKTKL (Pancreatic elastase EC 3.4.21.36)	α_{s2}-casein, gen. var. A-11P f(147–153)	3.0	10.84	865.08	1.98	−3.56	55.71	−0.929	0.998	0.5215	AMP	0.940
DTDYKKYL (Pancreatic elastase EC 3.4.21.36, Pepsin 1.3 EC 3.4.23.1, Pancreatic elastase EC 3.4.21.71)	β-lactoglobulin, gen. var. B f(96–103)	0.0	6.75	1045.16	3.3	−10.04	48.75	−1.788	1.000	0.4615	NAMP	0.556
KNTMEHV (Pancreatic elastase EC 3.4.21.36, Leukocyte elastase EC 3.4.21.37, Proteinase P1 (lactocepin) EC 3.4.21.96)	α_{s2}-casein, gen. var. A-11P f(1–7)	0.1	6.75	857.9	2.83	−14.33	41.43	−1.243	0.928	0.609	NAMP	0.029
NEEEYSI (Pancreatic elastase EC 3.4.21.36)	α_{s2}-casein, gen. var. A-11P f(48–54)	−3	3.67	882.8	3.66	101.71	55.71	−1.657	1.000	0.5665	NAMP	0.152
QNNDSTEYG (Pancreatic elastase EC 3.4.21.36)	α-lactalbumin, gen. var. B f(43–51)	−2	3,67	1026.9	4.39	42.26	0.00	−2.300	1.000	0.5875	NAMP	0.027
TKESPQTHYYA (Pancreatic elastase EC 3.4.21.36)	Lactoferrin f(84–94)	0.1	6.41	1324.4	2.68	156.75	9.09	−1.700	0.999	0.524	NAMP	0.027
PNSKEKYYG (Pancreatic elastase EC 3.4.21.36)	Lactoferrin f(517–525)	1.0	9.47	1085.18	3.03	13.17	0.00	−2.244	1.000	0.252	NAMP	0.873
LQGAVAKFFSASCVP (Prolyl oligopeptidase EC 3.4.21.26) Poor water solubility	Lactoferrin f(145–159)	1.0	8.97	1524.81	−0.58	54.95	84.67	0.980	0.785	0.2385	AMP	0.716
GQRDLLFKDSALGFLRIP (Prolyl oligopeptidase EC 3.4.21.26)	Lactoferrin f(294–311)	1.0	10.08	2046.41	1.52	20.89	113.89	0.028	0.659	0.4835	AMP	0.834
QEQNQEQP (Prolyl oligopeptidase EC 3.4.21.26), Thermolysin EC 3.4.24.27)	κ-casein, gen. var. A f(1–8)	−2	3.79	999.9	5.3	119.70	0.00	−3.263	0.976	0.5845	AMP	0.000

Table S2. *Cont.*

Sequence (Enzyme)	AMP Origin/Position	Net Charge	Isoelectric Point pH	Molecular Mass Da	Boman Index kcal/mol	Instability Index	Aliphatic Index	GRAVY	SVM	RFC	ANN	DAC
ADALNLDGGYIYTAGKCGLVPVLAE (V-8 proteaza EC 3.4.21.19)	Lactoferrin f(389–413)	−2.0	3.7	2523.89	−0.22	16.36	117.20	0.536	0.568	0.5215	NAMP	0.816
GYLAVAVVKKAN (Glutamyl endopeptidase II EC 3.4.21.82)	Lactoferrin f(432–443)	2.0	10.18	1232.49	−0.46	−0.31	130.0	0.733	0.893	0.4395	AMP	0.928
KKYKVPQL (Pepsin 1.3 EC 3.4.23.1, Pancreatic elastase EC 3.4.21.71)	α_{S1}-casein, gen. var. B-8P f(102–109)	3.0	10.45	1003.25	1.67	46.29	85.00	−1.262	0.952	0.509	NAMP	0.916
NVPGEIVESL (Pepsin 1.3 EC 3.4.23.1, Cathepsin G EC 3.4.21.20, Metridin EC 3.4.21.3, Pancreatic elastase II EC 3.4.21.71)	β-casein gen. var. B f(7–17)	−2	3.79	1056.1	0.47	50.75	136.00	0.340	0.586	0.516	NAMP	0.001
GRSAGWIIPMGIL (Pepsin 1.3 EC 3.4.23.1) Poor water solubility	Lactoferrin f(120–132)	1.0	11.04	1370.68	−0.82	52.79	127.69	0.923	0.171	0.564	AMP	0.645
FTKKTK (Thermolysin EC 3.4.24.27)	α_{s2}-casein, gen. var. A-11P f(147–152)	3.0	10.84	751.92	3.13	8.33	0.00	−1.717	1.000	0.5285	AMP	0.986
ANEEEYS (Thermolysin EC 3.4.24.27)	α_{s2}-casein, gen. var. A-11P (fr 47–53)	−3	3.67	840.8	4.11	101.71	14.29	−2.043	0.933	0.6535	NAMP	0.002
AGDDQG (Thermolysin EC 3.4.24.27)	Lactoferrin f(506–511)	−2	3.56	561.5	3.21	8.33	16.67	−1.583	0.624	0.551	NAMP	0.000
ESTEVF (Cathepsin G EC 3.4.21.20, (Pancreatic elastase II EC 3.4.21.71),	α_{s2}-casein, gen. var. A-8P f(152–147)	−2	3.80	710.7	2.09	72.53	48.33	−0.250	1.000	0.531	NAMP	0.005
PYPYYAK (Cathepsin G EC 3.4.21.20) Poor water solubility	κ-casein, gen. var. A f(57–63)	1.0	9.39	901.03	0.59	77.66	14.29	−1.314	0.999	0.3085	NAMP	0.719

Table S2. *Cont.*

Sequence (Enzyme)	AMP Origin/Position	Net Charge	Isoelectric Point pH	Molecular Mass Da	Boman Index kcal/mol	Instability Index	Aliphatic Index	GRAVY	SVM	RFC	ANN	DAC
TDAPSF (Chymase EC 3.4.21.39), Metridin EC 3.4.21.3)	α_{S1}-casein, gen. var. B-8P f(174–179)	−1	3.80	636.6	1.65	72.53	16.67	−0.333	0.901	0.5085	NAMP	0.011
AVAVVKKGSNF (Chymase EC 3.4.212.39, Metridin EC 3.4.21.3)	Lactoferrin f(94–104)	2.0	10.6	1119.33	0.13	−14.91	97.27	0.591	0.943	0.483	AMP	0.912
IIPMGILRPY (Chymase EC 3.4.21.39) Poor water solubility	Lactoferrin f(126–135)	1.0	9.84	1172.5	−0.79	47.87	156.0	0.980	0.371	0.595	AMP	0.837
VTAIANLKKCSTSPLLEACAF (Chymase EC 3.4.21.39) Poor water solubility	Lactoferrin f(666–686)	0.9	8.29	2180.62	0.00	43.29	107.14	0.748	0.685	0.917	AMP	0.852
PQRDMPIQAF (Chymase EC 3.4.21.39, Metridin EC 3.4.21.3)	β-casein gen. var. B f(181–190)	0	6.27	1202.3	2.26	72.20	49.00	−0.720	0.715	0.697	NAMP	0.018
EAGRDPY (Chymase EC 3.4.21.39), Metridin EC 3.4.21.3)	Lactoferrin f(66–72)	−1	4.37	806.8	3.97	8.57	14.29	−1.857	0.848	0.5785	NAMP	0.000
GTKESPQTHY (Chymase EC 3.4.21.39), Metridin EC 3.4.21.3)	Lactoferrin f(83–92)	0.1	6.75	1147.2	3.03	126.91	0.00	−1.96	0.718	0.625	NAMP	0.007
TTMPLW (Papain 3.4.22.2, Bromelain EC 3.4.22.4) Poor water solubility	α_{S1}-casein, gen. var. B-8P f(204–209)	0	5.19	747.9	−0.74	121.03	65.0	0.300	0.976	0.5275	NAMP	0.046
DMPIQA (Papain 3.4.22.2, Bromelain EC 3.4.22.4)	β-casein gen. var. B f(184–189)	−1	3.80	673.7	0.86	81.57	81.67	−0.067	0.768	0.525	NAMP	0.005
QPTTMA (Papain 3.4.22.2, Ficain EC 3.4.22.3, Leukocyte elastase EC 3.4.21.37, Bromelain EC 3.4.22.4) Poor water solubility	κ-casein, gen. var. A f(91–96)	0	5.52	647.7	1.08	61.00	16.67	−0.467	1.000	0.6035	NAMP	0.015

Table S2. *Cont.*

Sequence (Enzyme)	AMP Origin/Position	Net Charge	Isoelectric Point pH	Molecular Mass Da	Boman Index kcal/mol	Instability Index	Aliphatic Index	GRAVY	SVM	RFC	ANN	DAC
EERLHSMK (Bromelain EC 3.4.22.4)	α_{S1}-casein, gen. var. B-8P f(117–124)	0.1	6.86	1029.1	4.35	49.50	48.75	−1.712	0.717	0.5785	NAMP	0.001
SESTEDQA (Ficain EC 3.4.22.3, Bromelain EC 3.4.22.4)	α_{S1}-casein, gen. var. B-8P f(46–53)	−3	3.57	865.8	4.43	105.05	12.50	−1.812	0.998	0.637	NAMP	0.004
PQRDMPIQA (Ficain EC 3.4.22.3), Bromelain EC 3.4.22.4)	β-casein, gen. var. A-5P f(181–189)	0	6.27	1055.2	2.84	79.11	54.44	−1.111	0.827	0.6745	NAMP	0.010
EMPFPK (Ficain EC 3.4.22.3, Bromelain EC 3.4.22.4)	β-casein, gen. var. A^2-5P f(108–113)	0	6.94	747.91	1.17	145.77	0.00	−0.983	1.000	0.571	AMP	0.190
EPEQSL (Ficain EC 3.4.22.3)	β-lactoglobulin, gen. var. B f(112–117)	−2.0	3.13	701.73	2.94	174.73	65.00	−1.517	1.000	0.4695	NAMP	0.569
ESPQTHY (Ficain EC 3.4.22.3, Bromelain EC 3.4.22.4)	Lactoferrin f(86–92)	−0.9	5.24	860.8	3.3	189.14	0.00	−2.086	0.551	0.658	NAMP	0.004
PQLEIV (Leukocyte elastase EC 3.4.21.37, Prolidase L.lactis s.cremoris H61 EC 3.4.13.9)	α_{S1}-casein, gen. var. B-8P f(107–112)	−1	4.00	697.8	−0.25	58.38	178.33	0.650	0.945	0.553	NAMP	0.020
EKTTMPLW (Leukocyte elastase EC 3.4.21.37), Proteinase P1 (lactocepin EC 3.4.21.37)	α_{S1}-casein, gen. var. B-8P f(192–199)	0	6.10	1005.2	0.98	93.28	48.75	−0.700	1.000	0.644	NAMP	0.021
NEEEYS (Leukocyte elastase EC 3.4.21.37)	α_{s2}-casein, gen. var. A-11P f(48–53)	−3	3.67	769.7	5.1	117.0	0.00	−2.683	1.000	0.6795	NAMP	0.006

Table S2. *Cont.*

Sequence (Enzyme)	AMP Origin/Position	Net Charge	Isoelectric Point pH	Molecular Mass Da	Boman Index kcal/mol	Instability Index	Aliphatic Index	GRAVY	SVM	RFC	ANN	DAC
QETYKQEKNMA (Leukocyte elastase EC 3.4.21.37)	α_{s2}-casein, gen. var. A-11P f(17–27)	0.0	6.99	1369.52	3.72	76.85	9.09	−2.145	0.697	0.3575	NAMP	0.856
FTKKTKLTEEEKNRLNFLKKIS (Leukocyte elastase EC 3.4.21.37)	α_{s2}-casein, gen. var. A-11P f(147–168)	4.0	10.53	2696.19	3.06	29.53	70.91	−1.218	0.531	0.497	AMP	0.465
ITRINKKIEKFQS (Leukocyte elastase EC 3.4.21.37)	β-casein, gen. var. A^2-5P f(23–35)	3.0	10.83	1604.92	2.98	88.52	90.00	−0.915	0.492	0.454	AMP	0.739
LTLTDV (Leukocyte elastase EC 3.4.21.37) Poor water solubility	β-casein, gen. var. A^2-5P f(125–130)	−1	3.80	660.7	0.00	8.33	178.33	1.15	0.551	0.5625	NAMP	0.002
GWIIPMGILRPYLS (Leukocyte elastase EC 3.4.21.37) Poor water solubility	Lactoferrin f(124–137)	1.0	9.84	1616.01	−0.9	45.86	139.29	0.821	0.501	0.751	AMP	0.619
PPTVMF (Metridin EC 3.4.21.3) Poor water solubility	β-casein, gen. var. A^2-5P f(152–172)	0	5.96	690.8	−1.13	40.43	48.33	0.833	1.000	0.644	NAMP	0.053
RPTEGY (Metridin EC 3.4.21.3)	Lactoferrin f(428–433)	0	6.00	721.7	3.91	58.33	0.00	−2.0	1.000	0.4985	NAMP	0.025
DGGMVF (Metridin EC 3.4.21.3) Poor water solubility	Lactoferrin f(60–65)	−1.0	3.1	624.72	−0.42	28.90	48.33	0.767	0.998	0.644	AMP	0.001
APKHKEM (Pancreatic elastase II EC 3.4.21.71)	β-casein, gen. var. A^2-5P f(103–109)	1.1	8.64	840.0	2.62	69.91	14.29	−1.771	1.000	0.4915	AMP	0.020
KILDKVGINYWLAHKALCSEKLDQWLCEKL (Calpain EC 3.4.22.17)	α-lactalbumin, gen. var. B f(94–123)	1.0	8.26	3559.26	0.8	12.53	120.33	−0.113	0.691	0.5285	AMP	0.968
INYWLAHKALCSEKLDQWLCEKL (Glycyl endopeptidase EC 3.4.22.25)	α-lactalbumin, gen. var. B f(101–123)	0.0	7.0	2805.32	0.82	23.38	110.43	−0.183	0.554	0.556	AMP	0.763

Table S2. *Cont.*

Sequence (Enzyme)	AMP Origin/Position	Net Charge	Isoelectric Point pH	Molecular Mass Da	Boman Index kcal/mol	Instability Index	Aliphatic Index	GRAVY	SVM	RFC	ANN	DAC
APSITCVRRAFALECIRAIAEKKADAV TLDG (Glycyl endopeptidase EC 3.4.22.25)	Lactoferrin f(31–61)	0.9	8.29	3289.88	1.4	46.85	104.19	0.335	0.583	0.490	AMP	0.797
AVAKFFSASCVPCIDRQAYPNLCQLC KG (Glycyl endopeptidase EC 3.4.22.25) Poor water solubility	Lactoferrin f(149–175)	1.8	8.3	3033.62	0.61	25.65	76.79	0.375	0.903	0.9695	AMP	0.984
LEIVPN (Proteinase P1 (lactocepin) EC 3.4.21.96) Poor water solubility	α_{S1}-casein, gen. var. B-8P f(109–114)	−1	4.0	683.6	−0.07	58.38	178.33	0.650	0.556	0.535	NAMP	0.026
RDMPI Proteinase P1 (lactocepin) EC 3.4.21.96	α_{S1}-casein, gen. var. B-8P f(102-109)	0	5.84	630.7	3.27	95.88	78.00	−0.640	1.000	0.627	NAMP	0.015
ITRINKKIEKF (Proteinase P1 (lactocepin) EC 3.4.21.96)	β-casein, gen. var. A^2-5P f(23–33)	3.0	10.83	1389.71	2.71	62.85	106.36	−0.691	0.694	0.460	AMP	0.879
FMAIPPKKN (Proteinase P1 (lactocepin) EC 3.4.21.96)	κ-casein, gen. var. A f(105–113)	2.0	10.6	1045.32	0.63	40.80	54.44	−0.389	0.890	0.381	AMP	0.684
GAVAKFF (Proteinase P1 (lactocepin) EC 3.4.21.96) Poor water solubility	Lactoferrin f(147–153)	1.0	10.1	738.89	−1.28	−3.56	70.0	1.30	0.631	0.4245	AMP	0.597
ALFGKNGKNCPDKFCLFK (Proteinase P1 (lactocepin) EC 3.4.21.96)	Lactoferrin f(616–633)	2.9	9.71	2030.45	1.06	−5.79	48.89	−0.317	0.706	0.7655	AMP	0.987
PKHKEMPF (Prolidase L.lactis s.cremoris H61 EC 3.4.13.9)	β-casein, gen. var. A^2-5P f(104–111)	1.1	9.88	1013.23	2.15	117.35	0.00	−1.625	0.999	0.4065	NAMP	0.552

Fragments of known AMPs are marked in red.
